# Suppression of Endoplasmic Reticulum Stress by 4-PBA Protects Against Hyperoxia-Induced Acute Lung Injury *via* Up-Regulating Claudin-4 Expression

**DOI:** 10.3389/fimmu.2021.674316

**Published:** 2021-05-28

**Authors:** Hsin-Ping Pao, Wen-I. Liao, Shih-En Tang, Shu-Yu Wu, Kun-Lun Huang, Shi-Jye Chu

**Affiliations:** ^1^ The Graduate Institute of Medical Sciences, National Defense Medical Center, Taipei, Taiwan; ^2^ Department of Emergency Medicine, Tri-Service General Hospital, National Defense Medical Center, Taipei, Taiwan; ^3^ Division of Pulmonary and Critical Care Medicine, Department of Internal Medicine, Tri-Service General Hospital, National Defense Medical Center, Taipei, Taiwan; ^4^ Institute of Aerospace and Undersea Medicine, National Defense Medical Center, Taipei, Taiwan; ^5^ Department of Internal Medicine, Tri-Service General Hospital, National Defense Medical Center, Taipei, Taiwan

**Keywords:** 4-phenyl butyric acid, hyperoxia, acute lung injury, endoplasmic reticulum stress, claudin-4, oxidative stress

## Abstract

Endoplasmic reticulum (ER) stress that disrupts ER function can occur in response to a wide variety of cellular stress factors leads to the accumulation of unfolded and misfolded proteins in the ER. Many studies have shown that ER stress amplified inflammatory reactions and was involved in various inflammatory diseases. However, little is known regarding the role of ER stress in hyperoxia-induced acute lung injury (HALI). This study investigated the influence of ER stress inhibitor, 4-phenyl butyric acid (4-PBA), in mice with HALI. Treatment with 4-PBA in the hyperoxia groups significantly prolonged the survival, decreased lung edema, and reduced the levels of inflammatory mediators, lactate dehydrogenase, and protein in bronchoalveolar lavage fluid, and increased claudin-4 protein expression in lung tissue. Moreover, 4-PBA reduced the ER stress-related protein expression, NF-κB activation, and apoptosis in the lung tissue. In *in vitro* study, 4-PBA also exerted a similar effect in hyperoxia-exposed mouse lung epithelial cells (MLE-12). However, when claudin-4 siRNA was administrated in mice and MLE-12 cells, the protective effect of 4-PBA was abrogated. These results suggested that 4-PBA protected against hyperoxia-induced ALI *via* enhancing claudin-4 expression.

## Introduction

In critical pulmonary and cardiorespiratory disease, the delivery of oxygen to peripheral tissues is increased with supplemental oxygen treatment. However, prolonged exposure to very high concentrations of oxygen (≥ 50%) leads to an acute lung injury and acute respiratoryIntroduction distress syndrome (ALI/ARDS), and eventually death (1). ALI induced by exposure to supraphysiological concentrations of oxygen (hyperoxia) is characterized by capillary endothelial and alveolar epithelial cell damage, inflammatory cell infiltration, and non-cardiogenic pulmonary edema (2). Hyperoxia-induced acute lung injury (HALI) is caused by reactive oxygen species and the release of pro-inflammatory mediators that induces lung epithelial cell death *via* apoptosis or necrosis (3, 4). However, the molecular mechanisms behind HALI in this disease process have not been adequately understood.

The endoplasmic reticulum (ER) stress originates as a cascade of reactions called the unfolded protein response (UPR), which is activated in response to an accumulation of unfolded or misfolded proteins in the ER lumen (5). Three specialized ER-localized protein sensors are involved in UPR initiation: inositol-requiring enzyme 1α (IRE1α), double-stranded RNA-dependent protein kinase (PKR)-like ER kinase (PERK), and activating transcription factor 6 (ATF6), which are released from binding immunoglobulin protein (BiP; also known as glucose-regulated protein–78, or GRP78) during ER stress. GRP78 is an ER-resident chaperone, which associates with these three ER stress sensors and represses their activity (6, 7). During ER stress, GRP78 was released from these three proteins to activate UPR and modified downstream effectors, including phosphorylation α subunit of eukaryotic initiation factor-2 (eIF-2α), activating transcription factor 4 (ATF4), ATF6, and increasing CCAAT/enhancer-binding protein (C/EBP) homologous protein (CHOP) levels (8–10). In addition, ER stress can activate the NF-κB signaling, thereby promoting the production of various inflammatory mediators (11). ER stress has been contributed to the progression of the disease involving inflammation including respiratory diseases, diabetes, obesity, neurodegenerative diseases, inflammatory bowel diseases, and many metabolic diseases (5, 11–14). 4-Phenyl butyric acid (4-PBA) is commonly thought an “ER stress inhibitor” primarily as a chemical chaperone. The major mechanism for the action of 4-PBA is that the hydrophobic regions of the chaperone interact with exposed hydrophobic segments of the unfolded protein. This interaction protects the protein from aggregation, promotes the folding of proteins, and reduces ER stress. 4-PBA is an orally bioavailable, and low molecular weight fatty acid that has been approved by the Food and Drug Administration for clinical use to treat urea cycle disorders and hyperammonemia (15). 4-PBA has potential benefit for a wide variety of diseases like cancer, cystic fibrosis, thalassemia, spinal muscular atrophy as well as protein folding diseases such as type 2 diabetes mellitus, amyotrophic lateral sclerosis, Huntington disease, Alzheimer’s disease, and Parkinson disease (15–20). Moreover, ER stress has a role in developing HALI (21). However, the pathophysiology of ER stress in HALI is still elusive.

Claudins belong to a large family of integral membrane proteins that are essential components in the tight junction (TJ) formation and function. Different claudins with distinct roles modulated paracellular solutes transportation through adjacent epithelial cells. Claudin-3, claudin-4, and claudin-18 are predominantly expressed in the alveolar epithelium (22). Expression of claudin-4 protein well correlates with an alveolar barrier function in mice and human lungs (23, 24). Kage et al. reported that claudin-4 deficient mice had been shown to increase susceptibility to HALI (25). One study reported that *dermatophagoides farinae*-sensitized mice had increased ER stress and impaired airway epithelial barrier function which was associated with an exaggerated decrease of TJ proteins. In contrast, 4-PBA (an inhibitor of ER stress) inhibited the increase of ER stress and subsequently reversed the decrease of TJ proteins (26). Besides, 4‐PBA prevented the loss of TJ proteins by suppressing ER stress after spinal cord injury (27). Although it has been demonstrated that hyperoxia exposure decreases the protein expression of claudin-4 in the pulmonary epithelial barrier (28), the contribution of ER stress in HALI to pulmonary TJ barrier dysfunction is still not conclusive.

Therefore, the present study investigated whether 4-PBA reduced ER stress and enhanced the expression of the claudin-4 protein in a mouse model of HALI and then determined the role of claudin-4 protein in the beneficial effects of 4-PBA using small interfering RNA (siRNA). Similar studies were also performed in mouse lung epithelial cells exposed to hyperoxia.

## Methods

### Animal Model of Hyperoxia Exposure

Male C57BL/6J mice (8-10 weeks of age) were housed according to the National Institutes of Health Guidelines. All experiments were approved by the Animal Review Committee of National Defense Medical Center (approval number: IACUC-17-258). The room was regulated with 12 hours day/night cycle. Food and water were provided *ad libitum*. The mice were randomly assigned to the control group, hyperoxia group, and hyperoxia + 4-PBA group. 4-PBA (10 mg/kg per day; Sigma-Aldrich, St Louis, MO, USA) or saline was intraperitoneally administrated to mice at 0, 24, 48 and 72 h. The dose of 4-PBA in the present study was selected based on previous studies and our preliminary studies (20) (**Supplementary Figure 1**). The hyperoxia mice were exposed to more than 99% oxygen in an airproof chamber for 24, 48, and 72 hours, respectively, as described previously (3). The control mice were exposed only to room air.

### Survival Study

Mice treated with saline, or 4-PBA were continuously exposed to hyperoxia for evaluation of survival. The number of surviving mice was determined at 5-hours intervals until the last mouse died.

### Wet/Dry Lung Weight Ratio

The right upper lung lobe was excised at the end of the experiment. After the wet lung weight was determined, and then a part of the right upper lung lobe was dried in an oven at 60°C for 48 hours. The W/D weight ratio was calculated.

### Alveolar Fluid Clearance (AFC)

AFC was determined *in vivo* using an *in situ* model of mouse lung as previously described (29, 30). In brief, anesthetized mice were maintained at 37°C using a heating pad and lamp. During the experiment, the trachea was exposed and cannulated with an 18-gauge intravenous catheter. The lungs were inflated with 100% oxygen at continuous positive airway pressure of 7 cm H_2_O. Then, the instillate containing fluorescein isothiocyanate (FITC)-labeled albumin (Sigma-Aldrich, St. Louis, MO) was delivered to the lungs within 1 minute at a dose of 12.5 mL/kg. Alveolar fluid samples (100 μL) were collected 1 minute after instillation and at the end of the experiment (15 minutes later). The collected fluids were centrifuged at 3,000×g for 10 minutes, and the fluorescence activity in the supernatant was counted in duplicate. AFC was computed from the increase in alveolar fluid albumin concentration as follows:

$$\text{AFC }=\;\left({\text{C}}_{\text{f}}-{\text{C}}_{\text{i}}\right)/{\text{C}}_{\text{f}}\times 100,$$

where C_i_ and C_f_ represented the initial and final concentrations of FITC-albumin in the aspirate at 1 and 15 minutes, respectively, as assessed by the fluorescence activity measurements.

### Bronchoalveolar Lavage Analysis of Protein, Lactate Dehydrogenase (LDH), and Cytokine Contents

The left lung was lavaged twice with 0.5 mL of saline after the experiment. The bronchoalveolar lavage fluid (BALF) was centrifuged at 200×*g* for 10 minutes. The concentration of proteins in the supernatant was measured using a bicinchoninic acid protein assay kit (Pierce, Rockford, IL). Lactate dehydrogenase (LDH) activity in BALF was measured using the method as previously described (31). BALF tumor necrosis factor-α (TNF-α, catalog number: RTA00), Monocyte Chemoattractant Protein-1 (MCP-1, catalog number: RCN100), interleukin-6 (IL-6, catalog number: R6000B), and keratinocyte-derived chemokine (KC, catalog number: R6000B) levels were determined using a commercial mouse ELISA kit (R&D Systems Inc., Minneapolis, MN, USA).

### Malondialdehyde (MDA) and Protein Carbonyl Content in the Lung Tissue

MDA and protein carbonyl content were estimated as previously described (32). Briefly, the MDA of the supernatant was measured by absorbance at 532 nm and was expressed as nmol/mg protein. The protein carbonyl content was measured by the reaction with dinitrophenylhydrazine and was expressed as the concentration of carbonyl derivatives in the protein (nmol carbonyl/mg protein).

### Immunohistochemical Analysis of Lung Myeloperoxidase

Immunohistochemical staining to evaluate myeloperoxidase (MPO) was performed as described previously (33). Briefly, paraffin-embedded lung tissue sections were deparaffinized before antigen retrieval. The lung sections were incubated with a solution of 3% H_2_O_2_ in methanol for 15 min to block endogenous peroxidase. The slides were exposed to rabbit polyclonal antibody (anti-MPO, 1:100, Cell Signaling Technology, Danvers, MA, USA). The slides were then washed and incubated for 30 min with rat-specific horseradish peroxidase polymer anti-rabbit antibody (Nichirei Biosciences, Tokyo, Japan), and then horseradish peroxidase substrate was added for 3 min. The lung sections were then counterstained with hematoxylin.

### Western Blotting Analysis

The right lung samples and cell protein lysates were separated by 10% SDS polyacrylamide gel electrophoresis and immunoblots were developed as previously described (34). The membranes were blocked for 1 hours at room temperature with 5% nonfat milk and then incubated overnight at 4°C with the following antibodies: claudin-3 (Invitrogen, Carlsbad, CA, USA), claudin-4 (OriGene Technologies, Inc., Rockville, MD, USA), claudin-18 (Invitrogen), GRP78 (Abcam, Cambridge, MA, USA), phospho PERK (Taiclone, Taipei, Taiwan), phospho IRE1 (Abcam), ATF6 (Bioss Antibodies, Woburn, MA, USA), ATF4 (Bioss), GADD153 (CHOP, Santa Cruz Biotechnology, Dallas, TX, USA), B-cell lymphoma (Bcl)-2 (Santa Cruz Biotechnology), NF-κB p65, phospho-NF-κB p65, inhibitor of NF-κB (IκB)-α, cleaved caspase-3 and proliferating cell nuclear antigen (PCNA, Cell Signaling Technology), and β-actin (Sigma-Aldrich). All results were normalized to β-actin and expressed as the relative values to those for the control group.

### Immunofluorescence Detections for Claudin-4

Immunofluorescence staining for claudin-4 was performed as previously described (34). Lung tissue sections were deparaffinized, permeabilized using 0.1% Triton X-100, and blocked with 5% BSA before incubation with anti-claudin-4 (1:200, OriGene Technologies, Inc.) antibody overnight at 4°C. Fluorescein isothiocyanate-conjugated goat anti-rabbit IgG (1:200; Santa Cruz Biotechnology) was used as the secondary antibody for counterstaining. After washing in PBS and DAPI staining, slides were observed and images were captured using a fluorescence microscope (Leica DM 2500; Leica Microsystems GmbH, Wentzler, Germany).

### Cell Culture and Treatments

Mouse lung epithelial (MLE)-12 cells were cultured in DMEM/F-12 medium (Invitrogen) supplemented with 10% fetal bovine serum, penicillin (100 U/mL), and streptomycin (10 μg/mL) at 37°C and 5% CO_2_ in humidified air as described previously (34). These cells were exposed to hyperoxia (95% O_2_ and 5% CO_2_) for 72 hours. The cells were pretreated with vehicle, 4-PBA (0.3 mM), or claudin-4 siRNA (25 nM). The dose of 25 nM claudin-4 siRNA used in MLE-12 cell was determined by Western blot (**Supplementary Figure 2**). For claudin-4 siRNA transfection, MLE-12 cells were incubated for 24 hours and transfected with claudin-4 siRNA, using DharmaFECT™ 1 siRNA Transfection Reagent (Dharmacon Inc. Chicago, IL, USA). MLE-12 cells with non-targeting control siRNA treatment were used as negative controls. After 24 hours, the culture medium was replaced with the recommended medium. Forty-eight hours after claudin-4 siRNA treatment, MLE-12 cells were exposed to hyperoxia for 72 hours. Next, cells were lysed for protein extraction subjected to the western blot procedure. The supernatant was collected and assayed for KC using a mouse KC ELISA kit (R&D, Inc.). The experiments were performed in triplicates.

### siRNA Transfection *In Vivo*

100 μg of claudin-4 siRNA per mouse was administrated intratracheally in 50 μl. The intratracheal dose of 100 μg claudin-4 siRNA was determined by Western blot in the lung tissue (**Supplementary Figure 3**). Claudin-4 siRNA was sprayed directly into the mice lungs *via* the endotracheal route using a MicroSprayere aerolizer (Penn-Century, Philadelphia, PA, USA) as described previously (35). Forty-eight hours after injection, 4-PBA or saline was given intraperitoneally and then mice were exposed to hyperoxia for 72 hours. All the mice were sacrificed 72 hours after anesthesia, and the lung samples were collected.

### Electric Cell-Substrate Impedance Sensing (ECIS)

Epithelial barrier function was determined as previously described (36) using an ECIS system (Applied Biophysics, Troy, NY, USA). The ECIS with two 8W1E array plates (ibidi GmbH, Martinsried, Germany) served as a platform. The array holder was placed in a standard cell culture incubator (37°C, 100% humidity and 5% CO_2_). The array plates were equilibrated overnight with 400 μl of DMEM/F12 at cell culture conditions. Then 6×10^5^ MLE-12 cells were seeded on ECIS electrode arrays. After 20 hours of adherence, the arrays were affixed to the ECIS system and recorded with ECMS 1.0 software (CET, IA, USA). ECIS tracings expressed as transepithelial electric resistance (TER) are normalized to plateaued resistance values (subsequent values divided by initial values), and comparisons were made between 4-PBA (0.3 mM), claudin-4 siRNA (25 nM), and vehicle control-treated MLE-12 cells.

### Data Analysis

Statistical analyses were performed using GraphPad Prism 5 statistical software (GraphPad Software, San Diego, CA, USA). The data were presented as means ± SD, and multiple groups were evaluated using one-way ANOVA followed by a *post-hoc* Bonferroni test. Differences in survival were conducted using the Kaplan–Meier method with a log-rank test. *p-*value < 0.05 was considered statistically significant differences.

## Results

### 4-PBA Ameliorated HALI in Mice

**Figure 1:**
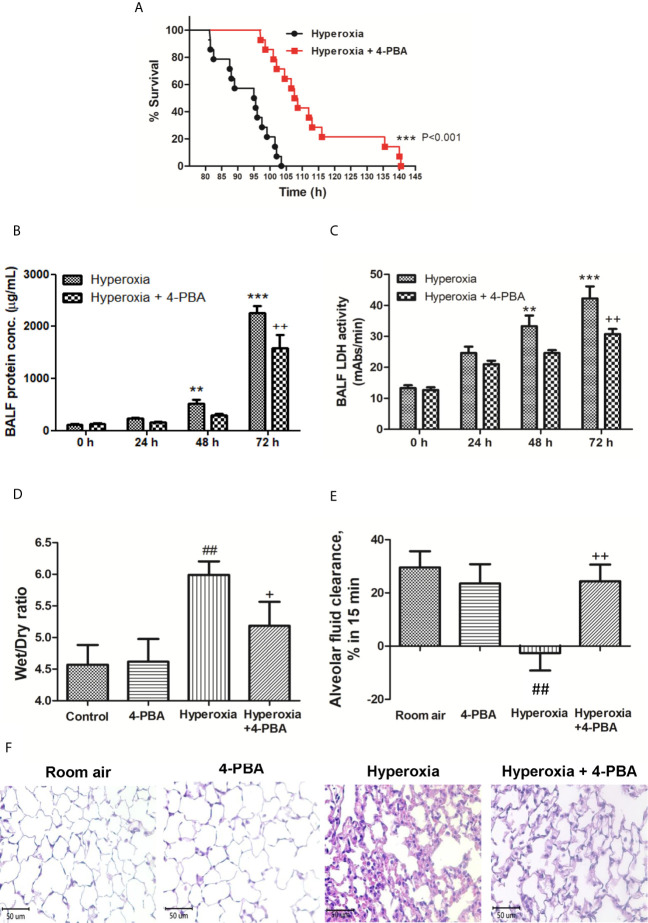
4-PBA prolonged survival rate, and improved lung edema and lung histopathology in mice with hyperoxia-induced lung injury. Survival was determined every 5 hours. The Kaplan–Meier survival curve was plotted and the difference in survival between the groups was significant (p < 0.001, log-rank test) **(A)**. Protein concentration in bronchoalveolar lavage fluid (BALF) **(B)**, BALF lactate dehydrogenase (LDH) activity **(C)**, and lung wet/dry ratio (W/D) **(D)** significantly increased in the hyperoxia group. Treatment with 4-PBA significantly attenuated the increase in these parameters. In addition, the 4-PBA increased AFC in mice exposed to hyperoxia 72 hours **(E)**. 4-PBA treatment significantly reduced thickening of the alveolar walls and neutrophil infiltration in the hyperoxia group. (representative results, Bar = 50 µm, hematoxylin, and eosin staining) **(F)**. Data are expressed as mean ± SD (6 mice per group). **p < 0.01, ***p < 0.001, compared with the 0 hour group; ^+^p < 0.05, ^++^p < 0.01, compared with the hyperoxia (72h) group; ^##^p < 0.01 compared with the room air group.

To investigate the protective effect of 4-PBA in mice with exposure to 100% O_2_, the survival was compared between the hyperoxia + 4-PBA group and the saline + hyperoxia group. All mice exposed to hyperoxia died within 105 h hours, whereas treatment with 4-PBA significantly prolonged survival in mice exposed to hyperoxia (**Figure 1A**). The protein and LDH levels in the BALF in mice with exposure to hyperoxia for 24, 48, and 72 hours were time-dependently increased (**Figures 1B, C**). The W/D ratio was significantly increased but AFC was significantly decreased in mice with exposure to hyperoxia for 72 hours when compared with the room air group (**Figures 1D, E**). Histologic evaluation of lung tissues in mice with exposure to hyperoxia for 72 hours showed distinct alveolar wall thickening and increased neutrophil infiltration in the interstitium and alveoli (**Figure 1F**). In contrast, treatment with 4-PBA significantly decreased protein and LDH levels in the BALF, and W/D ratio, increased AFC, and improved pathological change in lung tissue (**Figures 1B–F**).

### 4-PBA Suppressed Hyperoxia-Induced Increase of Inflammatory Mediator Production in BALF

**Figure 2:**
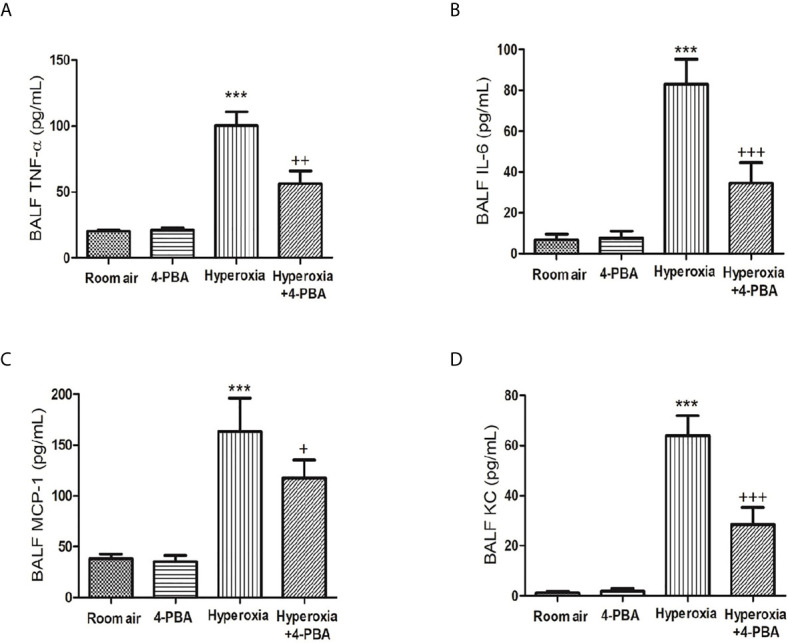
4-PBA alleviates hyperoxia-induced inflammatory mediator production in the bronchoalveolar lavage fluid (BALF). TNF-α **(A)**, IL-6 **(B)**, MCP-1 **(C)**, and KC **(D)** levels were analyzed by ELISA in the BALF. Data are expressed as mean ± SD (6 mice per group). ***p < 0.001, compared with the room air group; ^+^p < 0.05, ^++^p < 0.01, ^+++^p < 0.001 compared with the hyperoxia group.

To assess the anti-inflammatory effects of 4-PBA on HALI, TNF-α, IL-6, KC, and MCP-1 production in BALF were detected. The levels of TNF-α, IL-6, MCP-1 and KC were significantly increased after exposure to hyperoxia for 72 hours compared with the room air group (**Figures 2A–D**). However, 4-PBA-treated mice markedly reduced the level of these inflammatory mediators in BALF. These results indicate that 4-PBA may attenuate HALI by suppressing the inflammatory response.

### 4-PBA Attenuated Hyperoxia-Induced ROS Production

**Figure 3:**
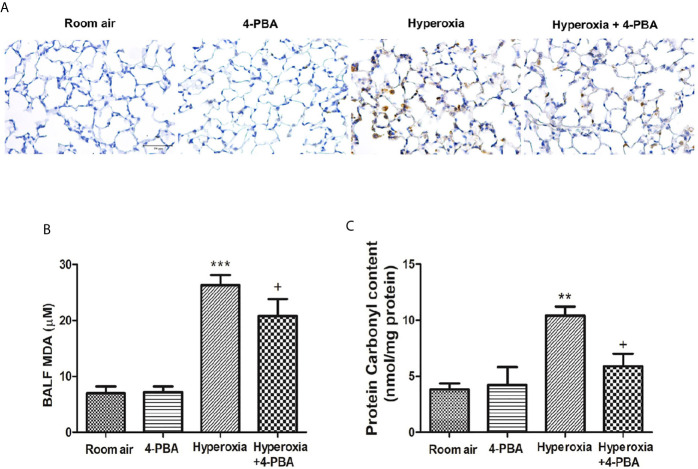
4-PBA attenuates hyperoxia-induced oxidative stress in lung tissue. Immunohistochemical staining (×200) of MPO in 72 hours after hyperoxia **(A)**. The MDA levels in the BALF **(B)**, and protein carbonyl contents **(C)** in lung tissues were analyzed by ELISA. Data are expressed as mean ± SD (6 mice per group). **p < 0.01, ***p < 0.001, compared with the room air group; ^+^p < 0.05 compared with the hyperoxia group.

The index for oxidative stress including MPO, MDA, and protein carbonyl was examined to evaluate the anti-oxidative activity of 4-PBA in mice with HALI. Hyperoxia induced significant increases in the numbers of MPO-positive cells, and the levels of MDA and protein carbonyl contents in the lung tissue (**Figures 3A–C**). However, 4-PBA significantly suppressed these increases.

### 4-PBA Inhibited NF-κB Signaling Pathway and Apoptosis in Mice With HALI

**Figure 4:**
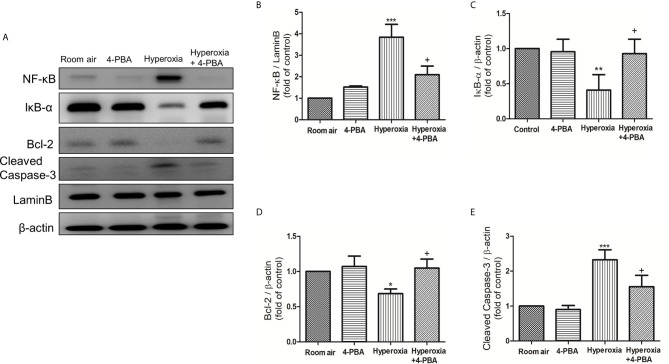
4-PBA suppresses NF-κB signaling pathway and apoptosis in mice with HALI. **(A)** Western blot analysis of NF-κB, IκB-α, Bcl-2 and cleaved caspase-3 expression in lung tissues. Relative expressions of NF-κB **(B)**, IκB-α **(C)**, Bcl-2 **(D)**, and cleaved caspase-3 **(E)** in lung tissues were shown. Data are expressed as mean ± SD (3 mice per group). *p < 0.05, **p < 0.01, ***p < 0.001, compared with the room air group; ^+^p < 0.05 compared with the hyperoxia group.

To investigate whether 4-PBA treatment can inhibit the NF-κB signaling pathway and apoptosis in HALI, the protein expressions of NF-κB, IκB-α, cleaved caspase-3, and Bcl-2 were measured by Western blotting. The reduction in cytoplasmic IκB-α after 72 hours of exposure to hyperoxia corresponded with an increase in nuclear p65, indicating activation of the NF-κB pathway (**Figures 4A–C**). The level of cleaved caspase-3 was significantly increased (**Figures 4A, E**), whereas the Bcl-2 was significantly decreased in the hyperoxia group compared to the room air group (**Figures 4A, D**). In contrast, 4-PBA treatment significantly increased IκB-α and Bcl-2 levels, and decreased NF-κB p65 and cleaved caspase-3 levels.

### 4-PBA Reduced ER Stress-Related Protein Expressions in Mice With HALI

**Figure 5:**
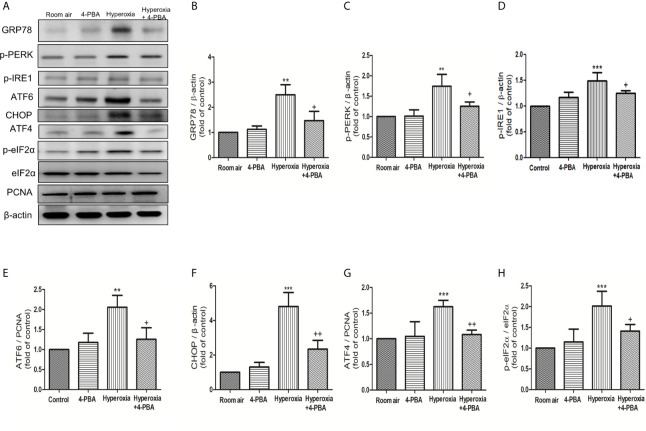
4-PBA alleviates hyperoxia-induced ER stress-related proteins in lung tissues. **(A)** Western blot analysis of GRP78, p-PERK, p-IRE1, ATF6, CHOP, ATF4, and p-elF-2 expression in lung tissues. Relative expressions of GRP78 **(B)**, p-PERK **(C)**, p-IRE1 **(D)**, ATF6 **(E)**, CHOP **(F)**, ATF4 **(G)**, and p-elF-2 **(H)** in lung tissues were shown. Data are expressed as mean ± SD (3 mice per group). **p < 0.01, ***p < 0.001, compared with the room air group; ^+^p < 0.05, ^++^p < 0.01 compared with the hyperoxia group.

To evaluate whether ER stress-related proteins are activated upon HALI, the protein levels of GRP78, p-PERK, p-IRE1, ATF-6, CHOP, ATF-4, and p-eIF2α were measured in lung tissues. Western blot analyses demonstrated that the levels of GRP78, p-PERK, p-IRE1, ATF-6, CHOP, ATF-4, and p-eIF2α in lung tissues were significantly increased after 72 hours hyperoxia exposure, compared with the room air group. The increase of ER stress-related proteins after 72hours hyperoxia exposure were markedly reduced by administration of 4-PBA (**Figures 5A–H**).

### 4-PBA Restored the Hyperoxia-Induced Disruption of Tight Junctions in Lung Tissue

**Figure 6:**
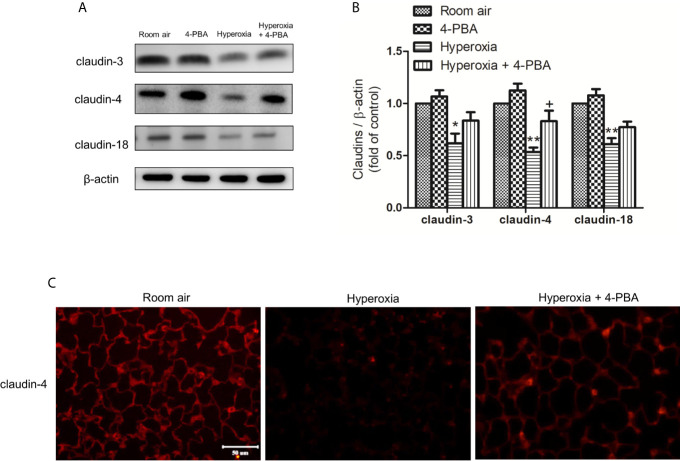
4-PBA enhances claudin-4 protein expression in mice with HALI. **(A)** Western blot analysis of claudin-3, claudin-4, and claudin-18 expression in lung tissues. **(B)** Relative expressions of claudins in lung tissues were shown. **(C)** Representative immunofluorescence staining of claudin-4 (red fluorescence). The results are expressed as the mean ± SD; n= 3. *p < 0.05 and **p < 0.01, compared with the room air group, ^+^p < 0.05 compared with the hyperoxia group.

To understand whether 4-PBA can regulate the expression of various claudin proteins in mice with HALI, claudin-3, claudin-4, and claudin-18 in the lung were determined. The result demonstrated that the levels of claudin-3, claudin-4, and claudin-18 in lung tissues were significantly decreased at 72 hours exposed to hyperoxia, compared with the room air group. The decrease of claudin-4 protein after hyperoxia exposure was significantly increased by administration of 4-PBA but not for claudin-3 and claudin-18 (**Figures 6A, B**). Immunofluorescence images from the room air group indicated that claudin-4 was localized in the alveolar epithelium cells showing continuous distribution. After 72 hours exposure to hyperoxia, claudin-4 showed discontinuous and decreased expression. Treatment with 4-PBA increased the staining intensity compared with the hyperoxia group, demonstrating retrieval of claudin-4 protein expression (**Figure 6C**).

### 4-PBA Protected Against Hyperoxia-Induced Epithelial Barrier Dysfunction in MLE-12 Cells *via* Enhancing Claudin-4 Expression

**Figure 7:**
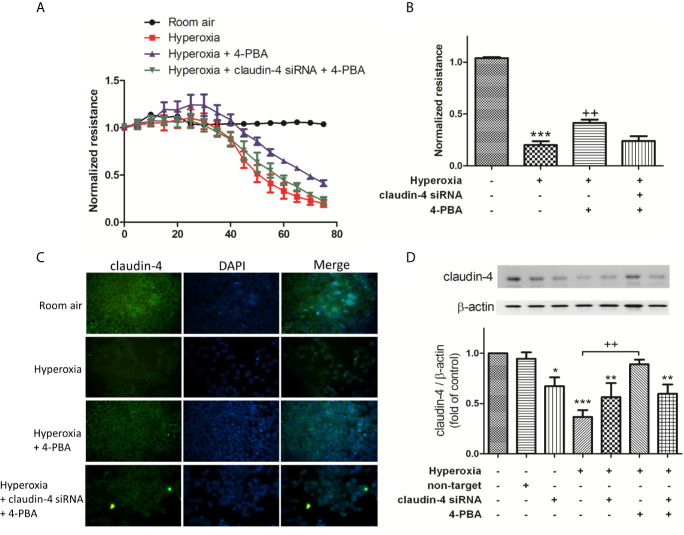
Claudin-4 knockdown abrogates the beneficial effects of 4-PBA on barrier function of MLE-12 cells. **(A)** Dynamic measurement of barrier function in MLE-12 cells subjected to room air, hyperoxia, hyperoxia + 4-PBA, and hyperoxia + claudin-4 siRNA + 4-PBA. Depicted plots were mean normalized resistance values with standard error from 3 repeated measures in each condition **(B)** Quantitation of normalized resistance 72 hours in each condition in MLE-12 cells **(C)** Representative immunofluorescence staining of claudin-4 (green fluorescence). **(D)** Representative Western blot and densitometry analysis of claudin-4. Data are mean ± SD, each experiment was performed at least independently in triplicate, *p < 0.05, **p < 0.01, ***p < 0.001 compared with the room air group, ^++^p < 0.01, compared with the hyperoxia group.

To verify the role of claudin-4 in the protective effect of 4-PBA, cells were transfected with siRNA targeting claudin-4. As shown in **Figures 7A, B**, MLE-12 decreased resistance after exposed to hyperoxia, as using ECIS assessment compared with the room air group. 4-PBA-treated MLE-12 significantly increased resistance after exposed to hyperoxia. But the protective effect of 4-PBA was blunted when claudin-4 was knockdown. The immunofluorescence staining showed that the decreased fluorescence intensity and disrupted distribution of claudin-4 was noted in the MLE-12 cells exposed to hyperoxia when compared with room air. 4-PBA failed to restore barrier function in claudin-4 siRNA -treated MLE12 exposed to hyperoxia (**Figure 7C**). Treatment with 4-PBA in MLE-12 increased claudin-4 protein expression in the hyperoxia group. But the increased claudin-4 protein expression induced by 4-PBA was abolished by claudin-4 knockdown (**Figure 7D**).

### The Protective Effects of 4-PBA in Attenuating KC Production, GRP78 and CHOP Protein Expression, and Apoptosis in Hyperoxia-Exposed MLE-12 Cells Were Blocked by Claudin-4 Knockdown

**Figure 8:**
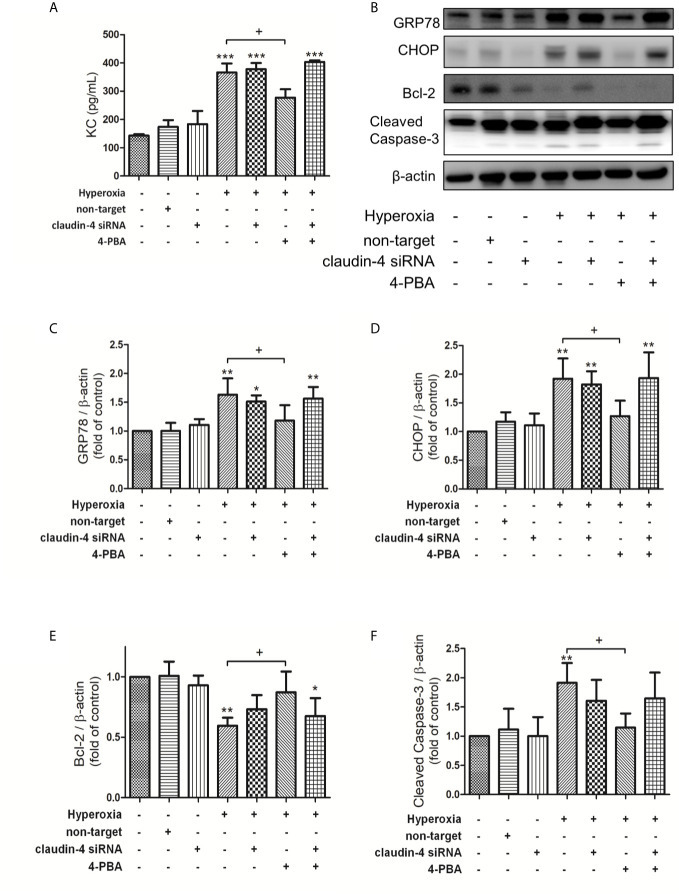
Claudin-4 knockdown blunts the beneficial effects of 4-PBA in KC production, GRP78 and CHOP protein expression, and apoptosis in hyperoxia-exposed MLE-12 cells. **(A)** KC in BALF was measured by ELISA. **(B)** Western blot analysis of GRP78, CHOP, Bcl-2, and cleaved caspase-3 expression in MLE-12 cells. Relative expressions of GRP78 **(C)**, CHOP **(D)**, Bcl-2 **(E)**, and cleaved caspase-3 **(F)** in MLE-12 cells were shown. Data are mean ± SDs, each experiment was performed at least independently in triplicate, *p < 0.05, **p < 0.01, ***p < 0.001 compared with the control group, ^+^p < 0.05, compared with the hyperoxia group.

In MLE-12 cells, 4PBA significantly attenuated the hyperoxia-induced increase in the level of KC, GRP78, and CHOP protein expression, and cleaved caspase-3 protein expression but decreased Bcl-2 protein expression. These beneficial effects of 4PBA were abolished in cells transfected with claudin-4 siRNA (**Figures 8A–F**).

### Claudin-4 Knockdown Abolishes the Beneficial Effect of 4-PBA in Mice With HALI

**Figure 9:**
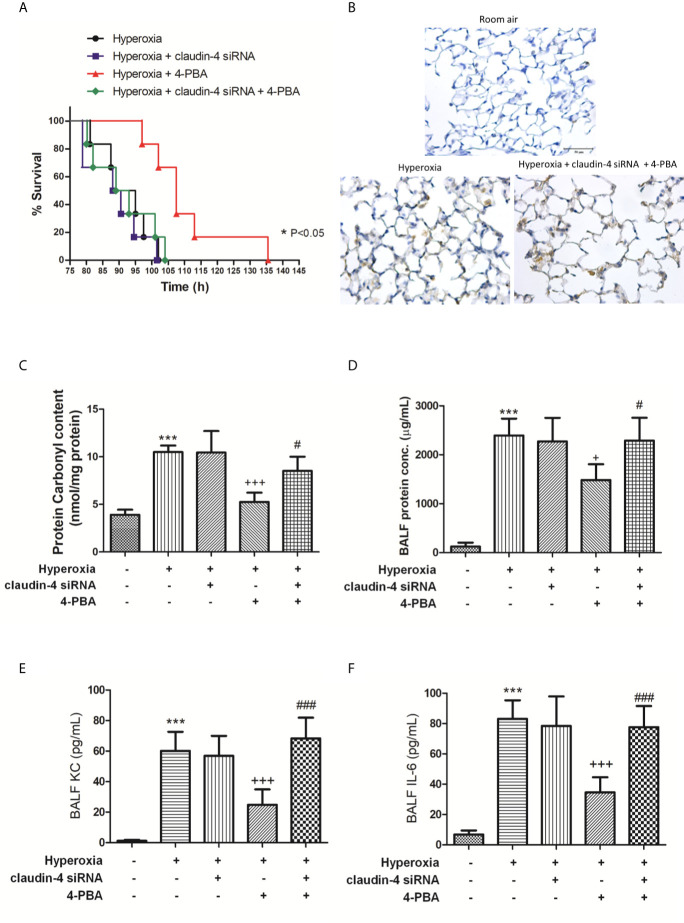
The beneficial effects of 4-PBA in mice with hyperoxia-induced lung injury were abolished by claudin-4 siRNA. Survival for each group was monitored during the observation period and plotted as Kaplan-Meier survival curves. The difference in survival between hyperoxia + 4-PBA and hyperoxia + claudin-4 siRNA + 4-PBA groups was significant (p < 0.05, log-rank test) **(A)**. Representative immunohistochemical staining images of MPO **(B)**. Protein carbonyl contents **(C)** in lung tissue and protein **(D)**, KC **(E)**, and IL-6 **(F)** levels in the BALF were measured by ELISA. The results are expressed as the mean ± SD; n=6. ***p < 0.001, compared with the control group; ^+^p < 0.05, ^+++^p < 0.001 compared with the hyperoxia group. ^#^p < 0.05, ^###^p < 0.001 compared with the hyperoxia + 4-PBA group.

When mice were treated with claudin-4 siRNA, the beneficial effect of 4-PBA in the survival of hyperoxia mice was abrogated (**Figure 9A**). Further, mice treated claudin-4 siRNA also abolished the effect of 4-PBA in decreasing pathological changes, carbonyl content in lung tissue, and protein, IL-6, and KC concentrations in BALF in hyperoxia-exposed mice (**Figures 9B–F**).

### Claudin-4 Knockdown Blocked the Effect of 4-PBA in ER Stress, NF-κB Signaling Pathway, and Apoptosis in Mice With HALI

**Figure 10:**
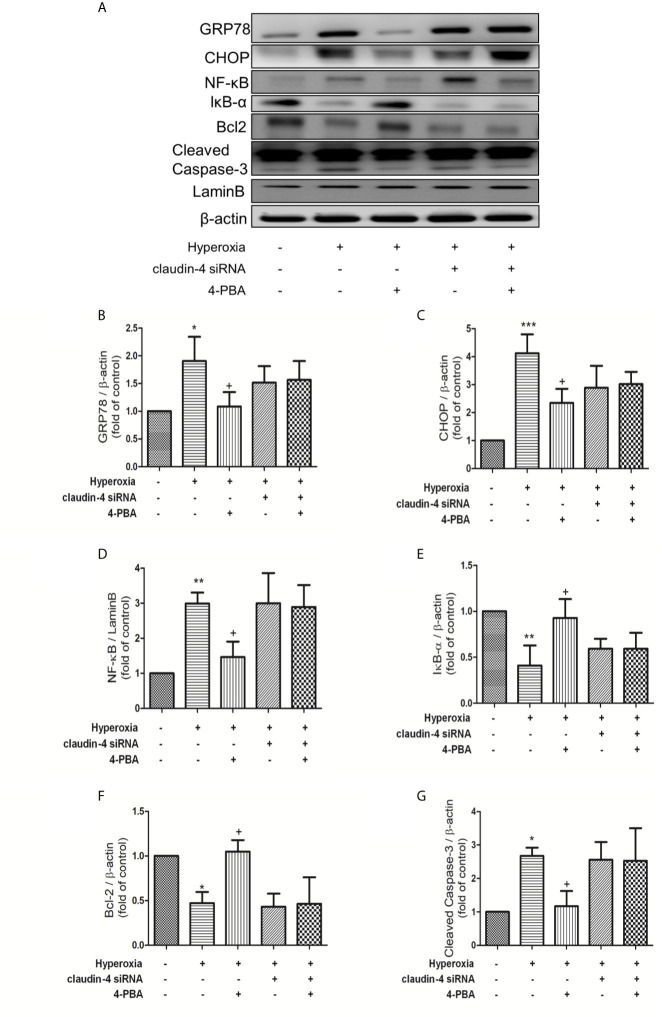
Claudin-4 knockdown attenuates the effect of 4-PBA in GRP78 and CHOP protein expression, NF-κB signaling pathway, and apoptosis in mice with hyperoxia-induced lung injury. **(A)** Western blot analysis of GRP78, CHOP, NF-κB, IκB-α, Bcl-2, and cleaved caspase-3 expression in lung tissues. Relative expressions of GRP78 **(B)**, CHOP **(C)**, NF-κB **(D)**, IκB-α **(E)**, Bcl-2 **(F),** and cleaved caspase-3 **(G)** in lung tissues were shown. The results are expressed as the mean ± SD; n=3. *p < 0.05, **p < 0.01, ***p < 0.001, compared with the control group; ^+^p < 0.05, compared with the hyperoxia group.

As shown in **Figure 10**, 4-PBA decreased GRP78, CHOP, NF-κB, and cleaved caspase-3 protein expressions, and increased IκB-α and Bcl-2 protein expressions. However, these beneficial effects of 4PBA disappeared when claudin-4 siRNA was employed in the mice (**Figures 10A–G**).

## Discussion

This study demonstrated that 4-PBA, a chemical chaperone, significantly improved multiple indices of HALI, such as prolonging survival, and decreasing AFC, lung edema, and disruption of tight junction proteins, production of pro-inflammatory cytokines, oxidative stress, the pulmonary neutrophil influx, and lung tissue damage. Furthermore, 4-PBA also inhibited hyperoxia-induced ER stress protein expressions, apoptosis, and NF-κB signaling pathways. Consistent with *in vivo* findings, 4-PBA treatment had a similar advantageous effect on MLE-12 epithelial cells exposed to hyperoxia. Importantly, 4-PBA enhanced claudin-4 protein expression in mice and MLE-12 cells exposed to hyperoxia. However, these protective effects of 4-PBA were abolished when claudin-4 was knockdown. These experiments indicate that 4-PBA may have potential benefits as adjuvant therapy for HALI and the protective mechanism was *via* enhancing claudin-4 expression.

The involvement of ER stress in pulmonary disorders including lung cancer, pulmonary fibrosis, pulmonary infection, cigarette smoke exposure, and asthma have been reported (37–41). ER stress contributes to impair hyperoxia-induced lung injury in the rat (42, 43). 4-PBA attenuates unfold protein aggregation and ER stress in LPS-induced lung inflammation (44). In this study, 4-PBA reduced the hyperoxia-induced up-regulation of GRP78, PERK, IRE1α, ATF4, ATF6, eIF2, and CHOP in both mice and MLE12. The results showed that 4-PBA was able to attenuate the pathologic changes associated with HALI. These findings suggest that ER stress is one of the crucial players during inducing and maintaining HALI.

Transmembrane and cytosolic proteins create a primary barrier to maintain lung fluid balance. The barrier function of all epithelia and endothelia is mainly provided by tight junction proteins. Claudins were integral membrane proteins found in tight junction that mainly provided the barrier function of all epithelia and endothelia (45). Hyperoxia exposure decreased claudin-4 protein expression that resulted in alveolar epithelial barrier dysfunction (18). Vyas‐Read et al. (46) demonstrated that the claudin composition of tight junctions in the pulmonary epithelium was affected by hyperoxia that increased rat sensibility to pulmonary edema and respiratory distresses. The results showed that hyperoxia impaired the expression of claudin-4 proteins and thereby increased the vascular permeability, and 4-PBA treatment significantly increased claudin-4 protein expression but not claudin-3 and claudin-18, which reduced the severity of lung injury. Moreover, in *in vitro* studies using MLE-12 epithelial cells exposed to hyperoxia, 4-PBA significantly attenuated the hyperoxia-induced epithelial barrier dysfunction and increased claudin-4 protein expression. However, the beneficial effect of 4-PBA was diminished by claudin-4 siRNA administration in mice and MLE-12 cells. These data suggested that the beneficial effect of 4-PBA in HALI was mediated by enhancing claudin-4 protein expression. Further study is needed to understand how 4-PBA increases the expression of claudin-4 in HALI.

It is clear that ER and oxidative stress are highly inter-related biological processes of exacerbating the inflammation which leads to pathophysiology in HALI (4). When ER protein folding is severely impaired in HALI, a large amount of ROS will be produced (4). High ROS levels can activate injured signaling pathways that aggravate inflammation, modify cellular signaling and function, leading to cell death. ER stress was also an important feature in epithelial cell dysfunction and death, both of which contributed to inflammation and disease. Harding et al. (47) have reported that the production of ROS has been linked to ER stress and the UPR. Further, ROS generation and ER stress are closely linked events of apoptosis (48). When the ER stress is lessened, cells will survive due to UPR-activated pro-survival signaling (48). Blocking ER stress with 4-PBA significantly decreased intracellular ROS generation and apoptosis (49–51). Therefore, ROS generation and ER stress accentuated each other through a positive feed-forward loop (48, 52). In the present study, administration of 4-PBA effectively reduced ER stress, MDA, protein carbonyl, and inflammatory response in HALI. Our results were also comparable with previous reports (50, 51). However, the exact mechanism needs further clarification.

A lot of evidence has demonstrated that ROS can disrupt epithelial and endothelial TJ proteins (53). Compromised tight junction barrier increases paracellular permeability and triggers a series of events including apoptosis and inflammatory response in the gastrointestinal tract, liver, kidney, lung, and brain (35, 53, 54). TJ barrier dysfunction and inflammation are closely related with each other, and proinflammatory cytokines contributes to the inflammatory cascade, TJ dysregulation, and apoptosis. Further, ROS can disturb ER protein folding, induce ER stress, and decrease the expression of TJs (49, 55). Subsequently, it can stimulate the UPR to cause apoptosis (49, 56). Previous studies also demonstrated the complex links between TJ proteins, cell death pathways, and inflammatory responses (56). In this study, 4-PBA effectively reduced oxidative and ER stress, the level of proinflammatory cytokines, and apoptosis but increased claudin 4 protein expression in HALI. Therefore, the integration of the TJ proteins, inflammatory signaling pathways, ROS, ER stress and apoptosis is important to the pathogenesis of a variety of diseases. However, the detailed molecular mechanisms in the complex network of interactions are still not elucidated. Further study will be required to understand the interplay.

NF-kB signaling pathway is critical for inflammatory response and associated with the production of various cytokines and chemokines (57). Under physiological conditions, NF-κB proteins are normally sequestered in the cytoplasm by its endogenous inhibitor, IκB. Upon activation, IκB phosphorylation triggers ubiquitin-dependent degradation and subsequently releases NF-κB which translocates to the nucleus and induces transcription of target genes (58). Previous studies have previously demonstrated that HALI promoted IκB degradation and NF-κB activation (29, 46). It had been previously shown that inhibition of NF-κB attenuated the stretch-induced increase in alveolar epithelial cell permeability (59). Kim et al. (45) showed that 4-PBA attenuated LPS-mediated NF-κB activation in the lungs. In this study, 4-PBA significantly suppressed IκB degradation and NF-κB activation that led to decreased production of proinflammatory cytokines and chemokines, such as TNF-α, IL-6, MCP-1, and KC, and reduced leukocyte infiltration in lung tissue. Moreover, in *in vitro* studies, 4-PBA also significantly inhibited NF-κB activation and the production of KC in MLE-12. Therefore, the anti-inflammatory effect of 4-PBA could be partly explained by its inhibition of NF-κB signaling and the consequent production of pro-inflammatory cytokines. However, these protective effects of 4-PBA were canceled by claudin-4 gene knockdown.

The present study focused on changes in the protein levels of claudin-4 by 4-PBA. Other types of intercellular junctions, such as occludin and zonula occludens (ZO) proteins also regulate tight junction assembly. Occludin-deficient mice are viable, indicating that occludin is not required for epithelia to form functional tight junctions. Further, barrier function in ZO-1/ZO-2-deficient Madin-Darby canine kidney cells is comparable to that of wild-type controls (60). All claudin family members have their various roles in modulating paracellular solutes transportation through adjacent epithelial cells. The effects of combinations of other claudins with claudin-4 in HALI were not clear (61). Further, the possibility that ER stress is involved in the oligomerization of these proteins under hyperoxia condition should be further investigated.

**Figure 11:**
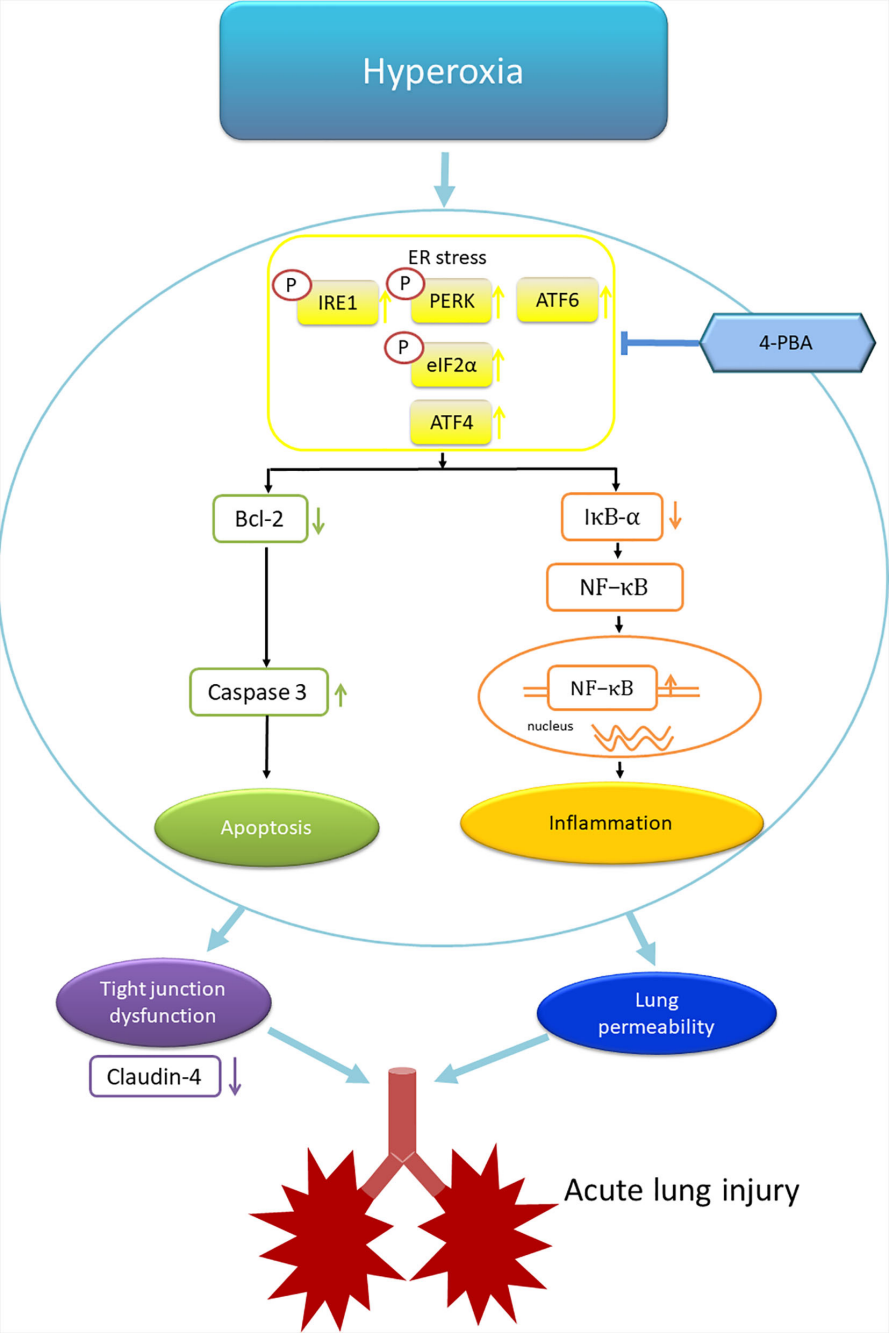
Schematic shows that hyperoxia triggers endoplasmic reticulum (ER) stress responses that lead to inflammation and apoptosis. The inflammation and apoptosis impair tight junction function and lung vascular permeability that induce acute lung injury. 4-PBA administration improves these phenomena. ATF4, activating transcription factor 4; ATF6, activating transcription factor 6; IRE1, inositol-requiring enzyme 1; PERK, protein kinase-like ER kinase. p-elF-2α, eukaryotic translation initiation factor 2α.

In summary, this study demonstrated that 4-PBA, an ER stress inhibitor, attenuated HALI by prolonging survival, decreasing lung edema, production of inflammatory cytokines, reactive oxygen species, apoptosis, and the activation of NF-κB and ER stress signaling (**Figure 11**). In addition, 4-PBA increased the TJ protein expression of claudin-4. The results of *in vitro* experiments with 4-PBA and MLE-12 cells exposed to hyperoxia confirmed the *in vivo* experiments. But these beneficial effects of 4-PBA were abolished when claudin-4 was knocked down. Further understanding of the physiological action of 4-PBA for tight junction protein is needed before being considered as a therapeutic option in HALI.

## Data Availability Statement

The raw data supporting the conclusions of this article will be made available by the authors, without undue reservation.

## Ethics Statement

The animal study was reviewed and approved by the Animal Review Committee of National Defense Medical Center (approval number: IACUC-17-258).

## Author Contributions

H-PP, K-LH and S-ET participated in research design. H-PP and S-YW conducted experiments. W-IL and S-YW performed data analysis. H-PP and S-JC contributed to the writing of the manuscript. All authors contributed to the article and approved the submitted version.

## Funding

This study was supported, in part, by grants MOST 106-2314-B-016-019 -MY3 and MOST 109-2314-B-016-028- from Ministry of Science and Technology, Taiwan, TSGH-D-110045, TSGH-D-109078, and TSGH-C107-054 from Tri-Service General Hospital, and MND-MAB-110-038, MAB-109-021, MAB-108-016, and MAB-107-011 from Ministry of National Defense-Medical Affairs Bureau, Taiwan.

## Conflict of Interest

The authors declare that the research was conducted in the absence of any commercial or financial relationships that could be construed as a potential conflict of interest.

## Supplementary Material

Supplementary Figure 1 Survival was determined every 5 hours. The Kaplan–Meier survival curve was plotted. Mice administered 4-PBA 10 mg/kg had a higher survival rate versus the other three groups (p <0.001, log-rank test).

Supplementary Figure 2 MLE-12 cells were transfected with varying doses of claudin-4 siRNA and immunoblotted against claudin-4 or β-actin (loading control).

Supplementary Figure 3 Two doses of claudin-4 siRNA were administrated intratracheally. These lung lysates were immunoblotted against claudin-4 or β-actin (loading control).
