# Supplementary figures and images for: Suppression of Endoplasmic Reticulum Stress by 4-PBA Protects Against Hyperoxia-Induced Acute Lung Injury via Up-Regulating Claudin-4 Expression
Source: Front Immunol. 2021 May 28;12:674316. doi: 10.3389/fimmu.2021.674316 (PMC8194262; doi:10.3389/fimmu.2021.674316)

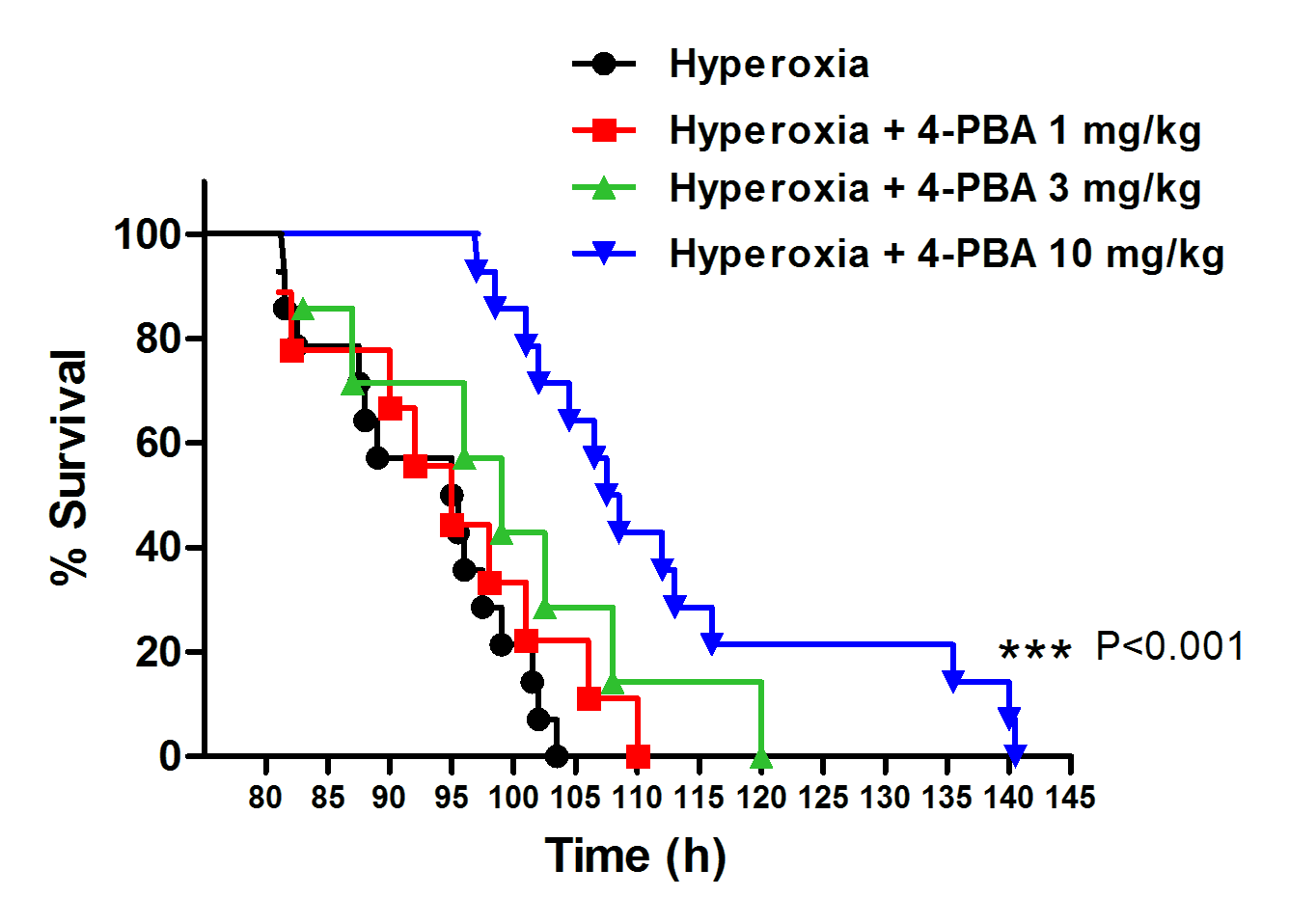

Supplement: Supplementary Figure 1 — Survival was determined every 5 hours. The Kaplan–Meier survival curve was plotted. Mice administered 4-PBA 10 mg/kg had a higher survival rate versus the other three groups (p <0.001, log-rank test). [file Image_1.tif]

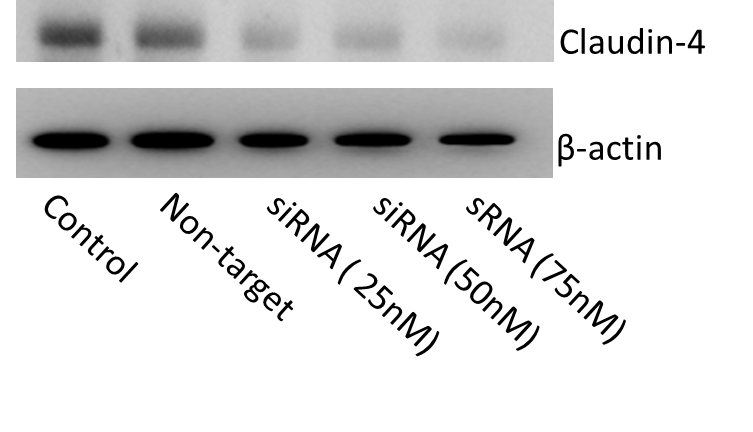

Supplement: Supplementary Figure 2 — MLE-12 cells were transfected with varying doses of claudin-4 siRNA and immunoblotted against claudin-4 or β-actin (loading control). [file Image_2.tif]

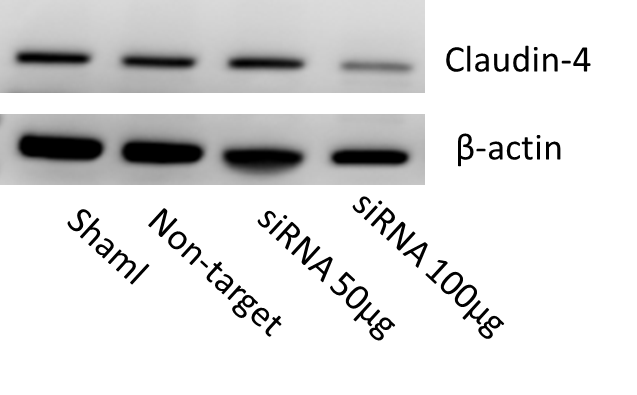

Supplement: Supplementary Figure 3 — Two doses of claudin-4 siRNA were administrated intratracheally. These lung lysates were immunoblotted against claudin-4 or β-actin (loading control). [file Image_3.tif]
